# Short-term outcomes of chest wall resections

**DOI:** 10.1007/s12055-025-01975-y

**Published:** 2025-05-31

**Authors:** Vishnu Santhosh Menon, Amita Sekhar Padhy, Rigved Nittala

**Affiliations:** Department of Surgical Oncology, Homi Bhabha Cancer Hospital & Research Centre, Visakhapatnam, 530053 Andhra Pradesh India

**Keywords:** Chest wall, Rib, Sternum, Sarcoma, Resection, Reconstruction

## Abstract

**Introduction:**

Chest wall resections (CWRs) pose a unique challenge for a thoracic surgeon by virtue of the complexities involved in maintaining anatomical integrity and functional dynamics of the region. We aimed at studying the outcomes of CWR from the thoracic surgery unit of a comprehensive cancer care centre located in a tier 2 city in India.

**Methods:**

This is a retrospective study of all CWRs from our centre, between 15 January 2019 to 15 January 2025. Patients were identified from a prospectively maintained surgical database and electronic medical records.

**Results:**

A total of 12 cases were identified who underwent CWR in the said duration, and the majority were for sarcoma (5/12, 41.6%). Rib resections were needed in 10/12 (83.3%) cases, with the 3rd rib (5/12, 41.6%) being the most commonly resected; and multiple rib resections were needed in 7/12 (58.3%) patients. Mesh repair was used in the majority of patients for reconstruction (10/12, 83.3%). No major perioperative morbidity was observed in any of the patients in the first 30 days of surgery.

**Conclusion:**

This study provides preliminary evidence for safe CWR being feasible at a low-volume thoracic surgical unit in India.

## Introduction

Chest wall resections (CWRs) represent a niche area of surgical expertise within the vast spectrum of surgical oncology and thoracic surgery [1]. The wide-ranging indications of CWR include surgery for primary chest wall tumours, secondary malignancies, as a part of en-bloc resection of thoracic masses, or other rare indications like infections and post-irradiation injury [2]. The focus areas from the point of view of the operating surgeon include ensuring radicality of resection with adequate bone and soft tissue margins, facilitating soft tissue cover and stabilization of the bony framework, while also ensuring good respiratory dynamics [3]. In India, few centres offer such complex surgical services, and most of this is centred in big cities and high-volume cancer centres [4]. In recent years, there has been a significant effort being put forth to develop better health infrastructure in even the smaller cities, and as a result, complex surgical procedures are being more readily attempted in even emerging and low-volume setups in tier 2 cities [5, 6]. The primary objective of this study was to explore the short-term outcomes of one such niche surgical procedure, i.e., CWR, being offered at our centre located in a tier 2 city in Eastern India.

## Methods

### Study design, population, and exclusion criteria

This study was a retrospective cohort analysis conducted at the thoracic surgery unit of a comprehensive cancer centre in eastern India. A prospectively maintained institutional database from the electronic medical records (EMR) was accessed to identify CWRs. The inclusion criteria included (1) resection of the chest wall for non-infective pathology at our centre, (2) treated with curative intent, (3) registered and operated between 15 January 2019 and 15 January 2025. The following exclusion criteria were applied: (1) recurrent disease at presentation, (2) prior surgery at an outside centre. The study adhered to ethical standards set by the institutional research committee and the Indian Council of Medical Research, in alignment with the principles of the 1964 Helsinki Declaration and subsequent amendments. Data was completely anonymized. This study was performed in accordance with the Strengthening the Reporting of Observational Studies in Epidemiology (STROBE) guidelines [7]. The primary objective of the study was to evaluate the spectrum of CWRs at our centre and their short-term outcomes.

### *Data* retrieval and statistical analysis

Clinical information, including patient demographics, pathological characteristics, surgical details, and postoperative outcomes of patients, were collected from the database and EMR.

We used Thoracic Surgical Morbidity and Mortality (TM&M) grading to report our postoperative surgical morbidity [8]. Data was analysed with IBM SPSS version 29. Descriptive statistics were used to analyse clinical, pathological, and radiological features and management protocols. Categorical variables were presented as numbers with percentages.

## Results

### Cohort characteristics (Table 1*)*

**Table 1:** Cohort characteristics

	**Median (range)**		**Mean ± SD**
**Age**	**41 (14–69)**		**40.3 ± 20.4**
	**Frequency**		**Percentage**
**Sex**			
Male	8		66.7%
Female	4		33.3%
**Epicentre**			
Soft tissue	6		50%
Ribs	4		33.3%
Lungs	2		13.3%
**Pathology**			
***Sarcoma***	5		41.6%
*Ewing* *sarcoma*	2		16.7%
*Synovial sarcoma*	1		8.3%
*Epithelioid sarcoma*	1		8.3%
*Spindle cell sarcoma*	1		8.3%
***Carcinoma***	3		25%
*Squamous cell carcinoma*	1		8.3%
*Adenocarcinoma*	2		16.7%
***Others***	4		33.3%
**ASA grade**			
*ASA1*	8		66.7%
*ASA2 or above*	4		33.3%
**Preoperative treatment**			
*Yes*	4		36.4%
*No*	8		66.7%
**Resection**			
***Rib resection***	10	83.3%	
*Single rib*	3	25%	
*Multiple ribs*	7	58.3%	
*Sternum*	1	8.3%	
***Lung resection***	4	33.3%	
*Wedge resection*	2	16.7%	
*Lobectomy*	2	16.7%	
*Diaphragm excision*	2	16.7%	
**Reconstruction**			
***Primary closure***	2	16.7%	
*Mesh*	10	83.3%	
*Mesh only*	6	50%	
*Mesh-bone cement*	4	33.3%	
**Margin negative resection**	12	100%	
**Postoperative course**			
*Minor complications* *(TM&M Grade I/II)*	4	33.3%	
*Major (TM&M Grade III or above) or perioperative mortality or readmission rate in 90 days*	0	0	
*Postoperative pulmonary complications*	2	16.7%	
*Wound-related complications*	1	8.3%	
**Adjuvant treatment**			
*Both postoperative chemotherapy and radiotherapy*	3	25%	
*Postoperative radiotherapy only*	2	16.7%	
*Postoperative chemotherapy only*	2	16.7%	

*SD*, standard deviation; *ASA grade*, American Society of Anesthesiologists risk grade; *TM&M grade*, Thoracic Surgical Morbidity and Mortality grade

During the said duration, 82 thoracic oncological resections were performed at our centre, and among them, 12 patients (14.6%), who underwent CWR, formed the study cohort. The median age of presentation was 41 years, ranging from 14 to 69 years, and three patients in this cohort were below 18 years of age. The majority were males (8/12, 66.7%) and right-sided (6/12, 50%). All patients were evaluated with contrast-enhanced computed tomography (CT) of the chest except for the patients of Ewing sarcoma, lung carcinoma, and breast carcinoma, who underwent an additional whole-body fluorodeoxyglucose positron emission tomography (FDG-PET) CT. All cases were discussed in a multi-disciplinary meeting and had a preoperative biopsy done. Sarcoma represented the most common pathology (5/11, 41.6%). The epicentre was the soft tissue of the chest wall in the majority of cases (6/12, 50%), followed by ribs (4/12, 33.3%) and intrathoracic sites, i.e., lungs (2/12, 16.7%). The majority were located along the anterior aspect (8/12, 66.7%), followed by the lateral aspect (3/12, 25%). Neoadjuvant treatment (NAT) was given to 4/12 (33.3%) patients, which included two patients with Ewing sarcoma, one patient with locally advanced breast carcinoma, and another patient with synovial sarcoma. We observed a response to NAT in the form of clinico-radiological partial response in all four patients. Surgical interventions were planned based on the baseline, or if applicable, post-chemotherapy CT scan, with a focus on identifying the tumour epicentre, intrathoracic spread, and defining the extent of resection.

**Fig. 1:**
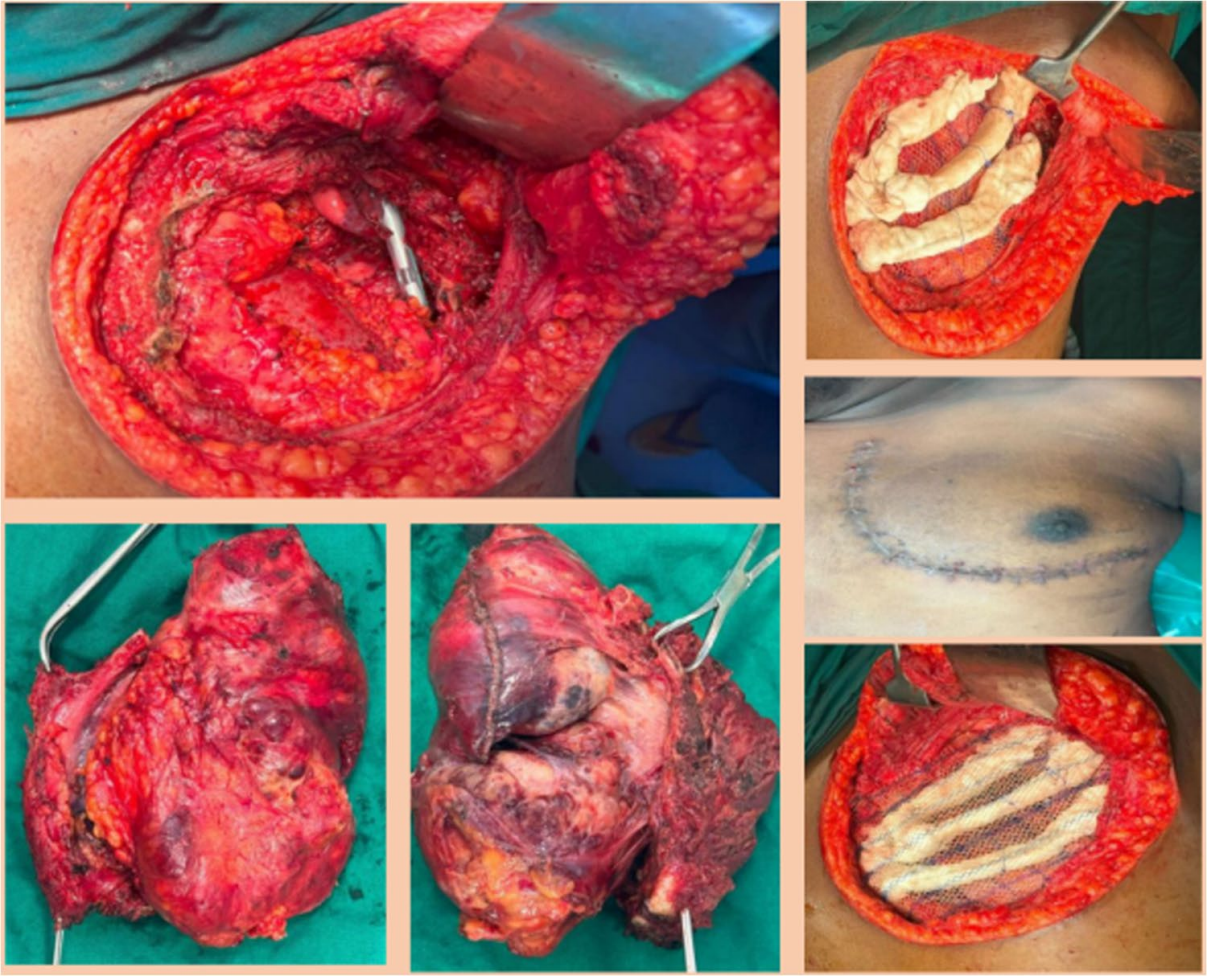
Chest wall reconstruction with mesh-bone cement neo-rib reconstruction

**Fig. 2:**
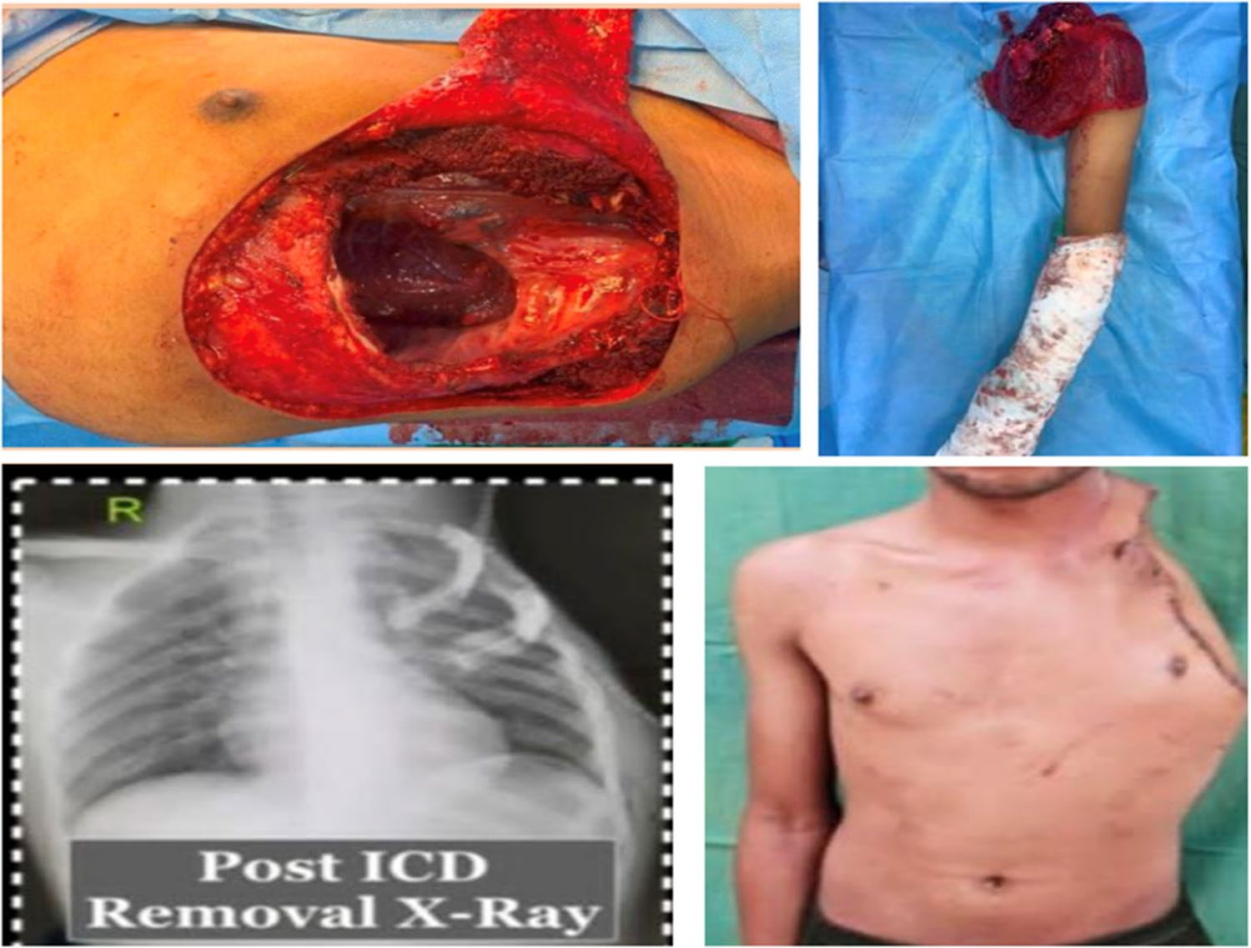
Left-sided chest wall spindle cell sarcoma of the antero-lateral chest wall infiltrating into the upper limb neuro-vasculature, requiring antero-lateral chest wall resection with left 1 st, 2nd, and 3rd rib excision, as well as left forequarter amputation with reconstruction using mesh-bone cement. This patient had an uneventful recovery post-surgery. In a clockwise direction, we can see the surgical defect after resection, the specimen image, the postoperative frontal profile of the patient, and the postoperative chest X-ray showing the neo-rib

The majority were American Society of Anesthesiologists Grade 1 risk patients (7/11, 58.3%). Sternal resection was required in one patient. The third rib was the most frequent rib excised (5/12, 41.6%) followed by the 6 th rib (3/12, 25%) and the 4 th rib (3/12, 25%). Multi-rib resections were required in 7/12 (58.3%) patients, with 4 patients (33.3%) requiring more than two rib resections. Diaphragm resection was required in two patients (2/12, 16.7%). Mesh-based reconstruction was used in 10/12 patients (83.3%) with mesh-only repair employed in 6/12 (50%). Rigid construction with mesh-bone cement (Neo-Rib) was needed in three patients (4/12, 33.3%) (Fig. 1). Lung resections were required in four patients, and two of these patients had lung primaries for which CWR was done as a part of en-bloc resection. One patient had spindle cell sarcoma of the chest wall infiltrating into subclavian vessels and the brachial plexus and invading into the thoracic outlet and required a planned left-sided forequarter amputation and CWR with reconstruction with mesh-bone cement (Fig. 2).

### Short-term outcomes and adjuvant treatment (Table 1)

The median duration of surgery was 140 min (ranging from 90 to 240 min). The median blood loss was 200 ml, ranging from 100 to 500 ml. No intraoperative complications were observed in any patients. In 66.7% (8/12), there were no surgical minor or major complications in the first 30 days of surgery. Postoperative pulmonary complications in the form of lung atelectasis and pneumonia were observed in 2/12 (16.7%) patients. We observed no wound-related complications in the first 30 days in any of the patients. Minor deviations from the routine postoperative course, including the need for blood transfusion and prolonged need for intravenous analgesia, were noted in 3/12 patients (25%). There were no readmissions or reinterventions in any of the patients in the first 30 days. Overall, TM&M Grade I/II postoperative complications were observed in 4/12 (33.3%) patients, and no patient had a major perioperative complication of TM&M Grade III and above.

Margin negative resection was achieved in all patients, with confirmation of the same in the final histopathological assessment. Adjuvant treatment decision was taken in the post-surgery Tumor Board discussion at the time of the first follow-up. Adjuvant chemotherapy alone was required in two patients, both of whom were lung carcinoma patients requiring en-bloc lung and CWR. Postoperative radiotherapy alone was required in two patients, both of whom were non-Ewing sarcomas. Postoperative adjuvant chemotherapy and radiotherapy were required in three patients, which included two patients with Ewing sarcoma and one patient with breast carcinoma.

### Late complications

We observed two patients needing re-surgeries due to complications of primary surgery. One patient, who underwent right 9 th rib fibrous dysplasia excision, required re-exploration following the development of liver herniation 4 years after surgery. This patient was managed with diaphragm plication by the double breasting technique. Another patient was a case of breast carcinoma who required CWR (total sternal resection) and reconstruction with dual mesh fixed into a plate of bone cement. She developed wound dehiscence after 4 months from the index surgery while being on adjuvant chemotherapy. This patient had a protracted course thereon, requiring multiple debridement and re-suturing. This patient required a rotation flap to fill the dehiscent area at 4 months.

### Use of video assistance (Fig. 3)

**Fig. 3:**
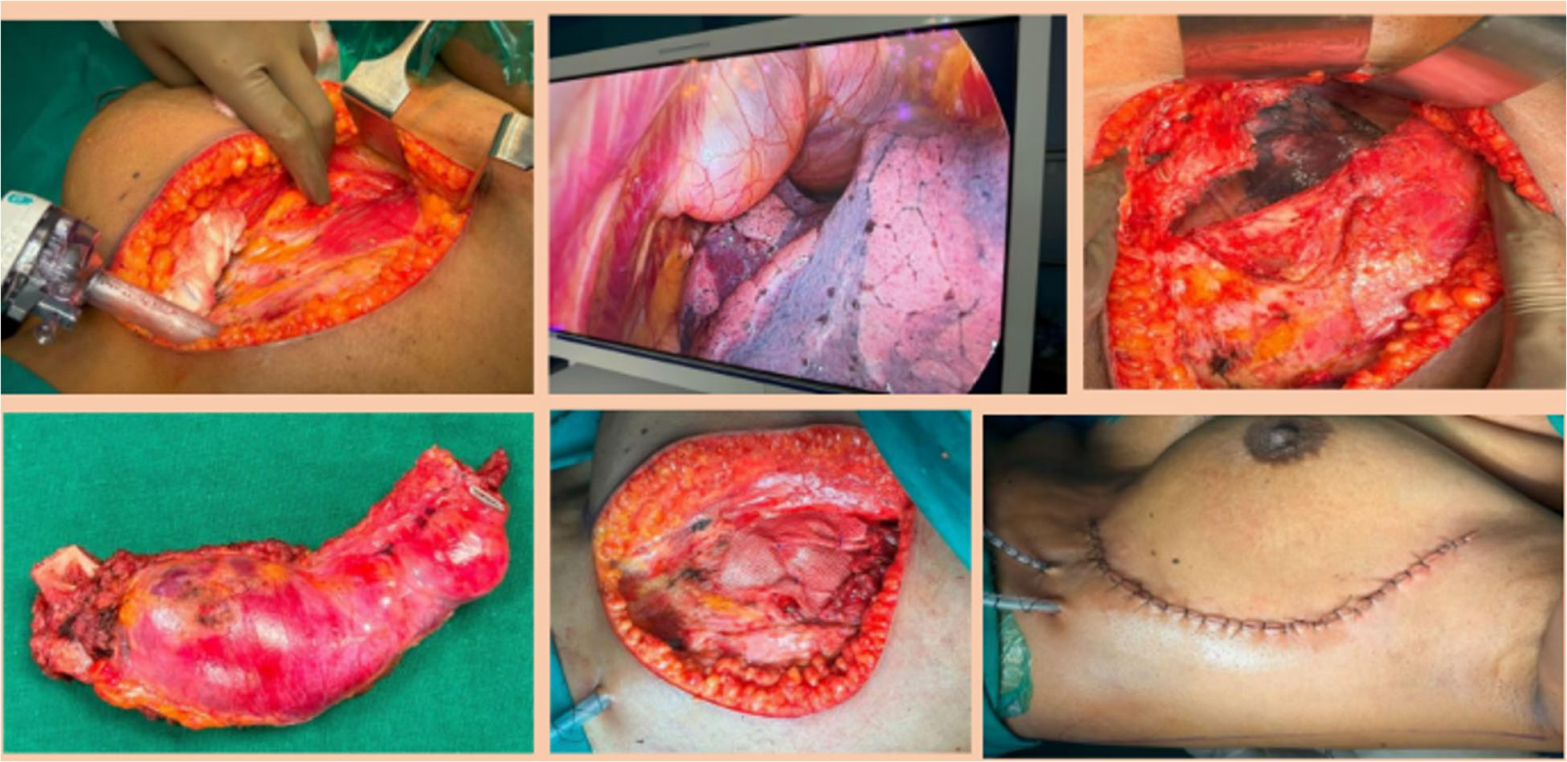
Video-assisted thoracoscopic-guided rib resection for a postero-lateral rib excision

We had employed video-assisted thoracoscopic surgical (VATS) approaches in two cases. This required raising of infra-mammary flaps and an incision along the lateral mammary fold (for cosmesis) and placement of thoracoscopic ports into two intercostal spaces below the rib to be excised. The benefit of this is three-fold, from our experience: (1) to allow better delineation of the site of the primary rib in cases in which the lesion was not palpable and (2) to allow the bone cut under vision and (3) better cosmesis with incisions hidden by body features like the infra-mammary crease, which is important in young females.

## Discussion

**Fig. 4:**
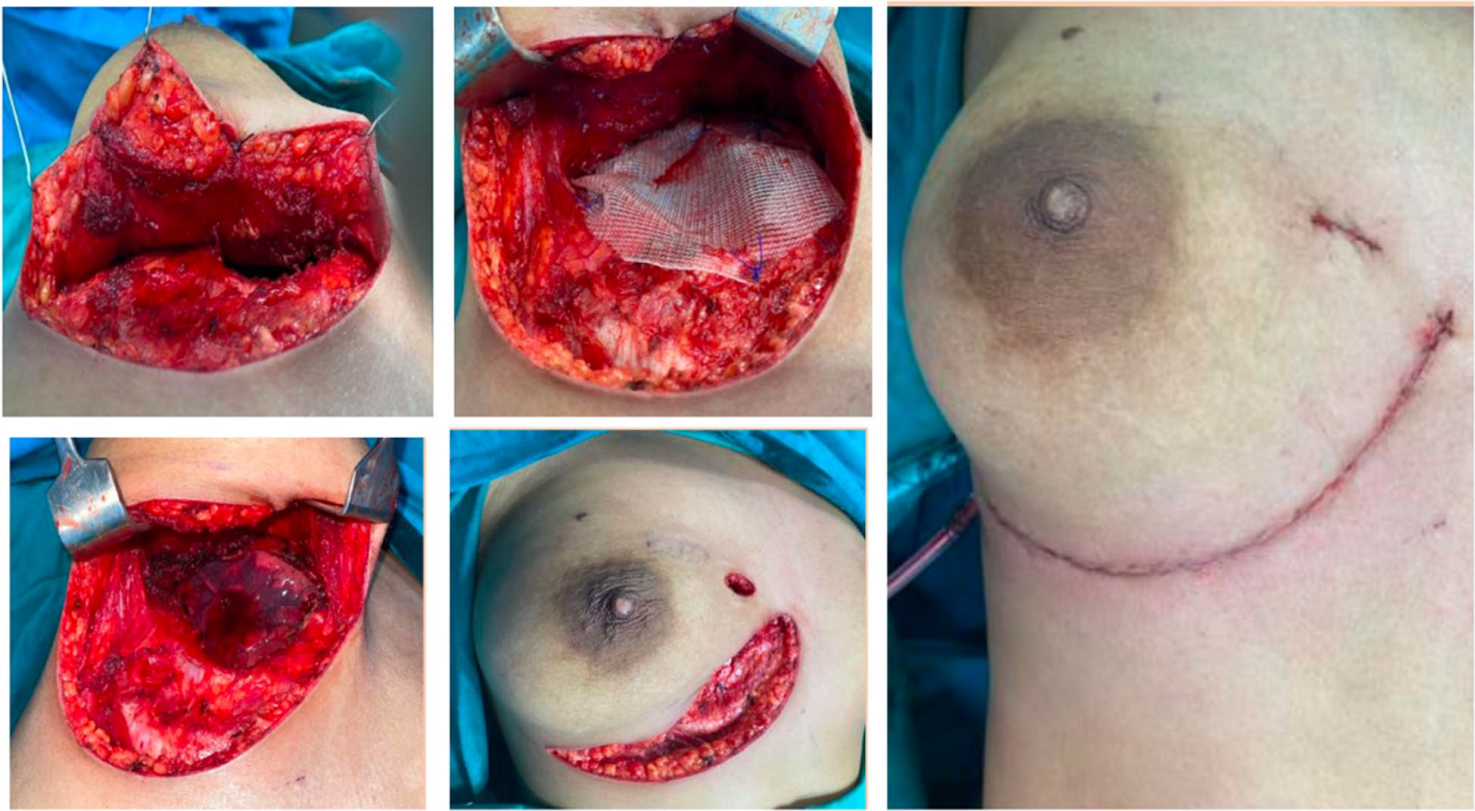
Anterior chest wall lesion in a 29-year-old lady who underwent right 3rd rib excision with reconstruction using mesh. The incision was placed in the infra-mammary crease, followed by elevation of flaps and excision of the rib and reconstruction. This planned incision ensured good cosmesis

Tensini described the first CWR with reconstruction using the latissimus dorsi flap in the early twentieth century. Since that time, we have progressed greatly in terms of the quality of resections and reconstructions [9]. The surgical planning requires a holistic approach, including sequencing of NAT, workup for surgery with incision planning, deciding on the extent of resection and reconstructive options, and postoperative rehabilitation and adjuvant therapy [1, 2, 10]. However, there is no consensus regarding when to reconstruct, but many thoracic surgeons agree that when more than 5 cm, or more than three ribs are resected, one should strongly consider reconstruction [10]. While the ease of surgical planning can improve with the use of computer-generated three-dimensional imaging and printing technologies, the cost factor and accessibility of the same are a few challenges which cannot be overlooked [11]. The ideal prosthetic material in CWR providing rigidity, malleability, inertness, and radiolucency is being actively researched upon and has led to the development of several reconstructive options over the years [12]. Titanium-based prosthetic bars are the newest options in the reconstructive ladder, but they are in no way ideal, nor are they easily accessible in resource-constrained setups like ours [13]. The use of mesh-bone cement neo-rib reconstruction was used in all of the patients in our cohort when requiring rigid reconstruction and has worked well as a cheaper and durable option. Apart from this, incision planning also forms a key challenge, particularly for anteriorly placed disease, and can become unsightly if not diligently executed. In our experience, planning an incision, which is well hidden by bodily features, like skin creases and folds, was found to improve cosmesis, particularly in anteriorly placed lesions (Fig. 4). Also, the use of VATS-guided rib resection is of particular importance, holding a lot of scope for future development by ensuring a minimally invasive approach and better cosmesis [14]. On a different tangent of radicality, we have performed a planned forequarter amputation along with CWR for a young patient with chest wall sarcoma involving upper limb neuro-vasculature. This highlights the need for a multi-disciplinary discussion and active patient participation in decision-making, especially when offering such extensive, morbid, yet curative resections. None of the patients in our cohort had any major postoperative complications, and there was no perioperative mortality (Table 1). However, the need for long-term follow-up, even in the patients who underwent rib resection for benign conditions, cannot be overlooked, as in our cohort, one patient developed complications 4 years after the initial surgery. These all underscore the need for evolving organizational measures in ensuring multi-disciplinary participation during all stages of treatment planning, including surgery. We have been successful in part not only due to these interdisciplinary collaborations, but also due to the efforts put into the frequent auditing of surgical outcomes, which had helped us to identify both the shortcomings and strengths in the system. As we dwell into an era of personalized medicine and focus on ensuring quality outcomes, there is a greater need for the standardization of such procedures and uniform reporting of outcomes. This is possible with multi-centre collaborations and requires strong organizational willpower, particularly from the national leaders of oncology and thoracic surgery. We hope our audit of CWRs encourages like-minded institutions to put forth their outcomes to explore means of expanding indications and options in chest wall reconstructions and standard reporting of outcomes.

### Limitations

The retrospective nature of the study, single-institution series, missingness of data, and lack of survival analysis are a few limitations of the study. We did not look into long-term oncological outcomes of the study cohort, as the majority of the patients were operated on in the past 2 years, and clinically meaningful follow-up could not be achieved to analyse the survival outcomes. However, for building a database of such complex and rare resections, we hope that through multi-institutional collaborations and under the guidance of organizations like the National Cancer Grid of India, we can put forth more robust data in the coming years [15].

## Conclusions

This study gives early evidence of CWR being safely performed at a resource constrained, low-volume thoracic cancer surgery unit in India. Post-resection complications can occur as late as 4 years from surgery, highlighting the need for long-term follow-up.

## Data Availability

The data that support the findings of this study are available on reasonable request from the corresponding author.

## References

[1] Novoa NM, Alcaide JLA, Hernadez MTG, Fuentes MG, Goni E, Jimenez L, et al. Chest wall—reconstruction: yesterday, today and the future. Shanghai Chest. 2019;3:15. 10.21037/shc.2019.02.02.

[2] Petrella F, Spaggiari L. Surgery of the chest wall: indications, timing and technical aspects. J Thorac Dis. 2020;12:1–2. 10.21037/jtd.2019.10.20. 32055416 10.21037/jtd.2019.10.20 PMC6995824

[3] Wang L, Yan X, Zhao J, Chen C, Chen C, Chen J, et al. Expert consensus on resection of chest wall tumors and chest wall reconstruction. Transl Lung Cancer Res. 2021;10:4057–83. 10.21037/tlcr-21-935. 35004239 10.21037/tlcr-21-935 PMC8674598

[4] Yendamuri S. Thoracic surgery in India: challenges and opportunities. J Thorac Dis. 2016;8:S596-600. 10.21037/jtd.2016.05.08. 27651933 10.21037/jtd.2016.05.08 PMC5009067

[5] Barik D, Thorat A. Issues of unequal access to public health in India. Front Public Health. 2015;3:245. 10.3389/fpubh.2015.00245. 26579507 10.3389/fpubh.2015.00245 PMC4621381

[6] Gopal KM. Increasing accessibility of hospitals in tier 2 and 3 cities through private participation. 2021; 10.13140/RG.2.2.35234.30402.

[7] von Elm E, Altman DG, Egger M, Pocock SJ, Gøtzsche PC, Vandenbroucke JP. The Strengthening the Reporting of Observational Studies in Epidemiology (STROBE) statement: guidelines for reporting observational studies. Int J Surg. 2014;12:1495–9.25046131

[8] Seely AJ, Ivanovic J, Threader J, Al-Hussaini A, Al-Shehab D, Ramsay T, et al. Systematic classification of morbidity and mortality after thoracic surgery. Ann Thorac Surg. 2010;90:936–42. 10.1016/j.athoracsur.2010.05.014. 20732521 10.1016/j.athoracsur.2010.05.014

[9] Ferraro P, Cugno S, Liberman M, Danino MA, Harris PG. Principles of chest wall resection and reconstruction. Thorac Surg Clin. 2010;20:465–73. 10.1016/j.thorsurg.2010.07.008. 20974430 10.1016/j.thorsurg.2010.07.008

[10] Geissen NM, Medairos R, Davila E, Basu S, Warren WH, Chmielewski GW, et al. Number of ribs resected is associated with respiratory complications following lobectomy with en bloc chest wall resection. Lung. 2016;194:619–24. 10.1007/s00408-016-9882-3. 27107874 10.1007/s00408-016-9882-3

[11] Leonardi B, Carlucci A, Noro A, Bove M, Natale G, Opromolla G, et al. Three-dimensional printed models for preoperative planning and surgical treatment of chest wall disease: a systematic review. Technologies. 2021;9:1–11. 10.3390/technologies9040097.

[12] Sanna S, Brandolini J, Pardolesi A, Argnani D, Mengozzi M, Dell’Amore A, et al. Materials and techniques in chest wall reconstruction: a review. J Vis Surg. 2017;3:95. 10.21037/jovs.2017.06.10. 29078657 10.21037/jovs.2017.06.10 PMC5638032

[13] Tamburini N, Grossi W, Sanna S, Campisi A, Londero F, Maniscalco P, et al. Chest wall reconstruction using a new titanium mesh: a multicenters experience. J Thorac Dis. 2019;11:3459–66. 10.21037/jtd.2019.07.74. 31559051 10.21037/jtd.2019.07.74 PMC6753435

[14] Hirji SA, Dezube A, Philips W, Balderson SS, Kara HV, D’Amico TA. Video-assisted thoracic surgery technique for chest wall resection. Operat Tech Thor Cardiovasc Surg. 2022;27:345–58. 10.1053/j.optechstcvs.2022.03.004.

[15] Pramesh CS, Badwe RA, Sinha RK. The national cancer grid of India. Indian J Med Paediatr Oncol. 2014;35:226–7. 10.4103/0971-5851.142040. 25336795 10.4103/0971-5851.142040 PMC4202620

